# Distribution of *Lutzomyia longipalpis* Chemotype Populations in São Paulo State, Brazil

**DOI:** 10.1371/journal.pntd.0003620

**Published:** 2015-03-17

**Authors:** Claudio Casanova, Fernanda E. Colla-Jacques, James G. C. Hamilton, Reginaldo P. Brazil, Jeffrey J. Shaw

**Affiliations:** 1 Superintendência de Controle de Endemias, Secretaria de Estado da Saúde, Mogi Guaçu, São Paulo, Brazil; 2 Departamento de Biologia Animal, Instituto de Biologia, UNICAMP, Campinas, São Paulo, Brazil; 3 Centre for Applied Entomology and Parasitology, School of Life Sciences, Keele University, Keele, United Kingdom; 4 Laboratório de Doenças Parasitárias, Instituto Oswaldo Cruz, Rio de Janeiro, Brazil; 5 Departamento de Parasitologia, Instituto de Ciências Biomédicas, Universidade de São Paulo, São Paulo, Brazil; Lancaster University, UNITED KINGDOM

## Abstract

**Background:**

American visceral leishmaniasis (AVL) is an emerging disease in the state of São Paulo, Brazil. Its geographical expansion and the increase in the number of human cases has been linked to dispersion of *Lutzomyia longipalpis* into urban areas. To produce more accurate risk maps we investigated the geographic distribution and routes of expansion of the disease as well as chemotype populations of the vector.

**Methodology/Principal Findings:**

A database, containing the annual records of municipalities which had notified human and canine AVL cases as well as the presence of the vector, was compiled. The chemotypes of *L*. *longipalpis* populations from municipalities in different regions of São Paulo State were determined by Coupled Gas Chromatography – Mass Spectrometry. From 1997 to June 2014, *L*. *longipalpis* has been reported in 166 municipalities, 148 of them in the Western region. A total of 106 municipalities were identified with transmission and 99 were located in the Western region, where all 2,204 autochthonous human cases occurred. Both the vector and the occurrence of human cases have expanded in a South-easterly direction, from the Western to central region, and from there, a further expansion to the North and the South. The (*S*)-9-methylgermacrene-B population of *L*. *longipalpis* is widely distributed in the Western region and the cembrene-1 population is restricted to the Eastern region.

**Conclusion/Significance:**

The maps in the present study show that there are two distinct epidemiological patterns of AVL in São Paulo State and that the expansion of human and canine AVL cases through the Western region has followed the same dispersion route of only one of the two species of the *L*. *longipalpis* complex, (*S*)-9-methylgermacrene-B. Entomological vigilance based on the routes of dispersion and identification of the chemotype population could be used to identify at-risk areas and consequently define the priorities for control measures.

## Author Summary

Information on the geographical distribution, dispersal mechanisms and dispersion route of insect-borne diseases can help to identify ongoing transmission areas, new risk areas and guide surveillance and control activities. *Lutzomyia longipalpis*, the principal vector of American visceral leishmaniasis disease in the Americas, is a group of closely related species that can be separated according to the type of pheromone produced by male individuals. It is still unclear how many members there are in this complex, how they are related and if some are more important vectors than others. In the present study, we show that the expansion of human visceral leishmaniasis in the state of São Paulo, Brazil, has followed the same dispersion route of only one of the two related species of *L*. *longipalpis* found in the state. The maps that we produced in the present study will allow us to determine risk areas for the occurrence of human visceral leishmaniasis, and reinforces our hypothesis that within São Paulo state these chemotype populations could have different biological capacities to act as a vector.

## Introduction

Recording the geographic distribution and identifying the possible routes of expansion of both arthropod-borne diseases and their associated vectors is essential information for surveillance as well as the execution and elaboration of control strategies [1].

In Brazil, the expansion of the geographic range of *Lutzomyia longipalpis* (Lutz & Neiva), the principal vector of *Leishmania* (*Leishmania*) *infantum chagasi* (Cunha & Chagas), and its adaptation to domiciliary habitats in the urban areas throughout Brazil has resulted in an increase in the incidence of both canine and human visceral leishmaniasis (VL) in the last 25 years [2–6]. According to the Brazilian Ministry of Health, in the period from 2009 to 2011, there were 251 municipalities classified as having moderate (mean number of human cases > = 2.4 and < 4.4) or intense (mean number of human cases > = 4.4) VL transmission in the country [7].

Before 1998, São Paulo State was considered free of autochthonous cases of this zoonotic disease and records of the vector’s presence were restricted to some rural areas of municipalities in the Northeast region of the state [8]. Two human cases had been reported in Greater São Paulo, but possible reservoirs and vectors were not described [9]. The first record of *L*. *longipalpis* in an urban area in São Paulo State was in 1997 from Araçatuba in the West of São Paulo State [8]. Canine and autochthonous human cases occurred in the same municipality in 1998 and 1999 respectively [10]. Since then, the appearance of *L*. *longipalpis* in urban areas of other municipalities has been linked to an increase in both canine and human visceral leishmaniasis within the State [11, 12]. From 1999 to April 2013, São Paulo State recorded 2204 autochthonous human cases of disease, with 192 deaths [13]. In São Paulo State, 18 municipalities were classified as having moderate or intense transmission in the period from 2010 to 2012 [11].

Based on genetic and behavioural studies it is generally accepted that *L*. *longipalpis* is a species complex, but it is unclear how many members there are and how they are related [14, 15]. Chemical, behavioural and ecological analysis of male produced sex pheromones suggests that *L*. *longipalpis* is a complex of at least four different, reproductively isolated members [16–19]. In Brazil two of these are represented by members where the males produce either 3-methyl-α-himachalene [20], a novel bicyclic methylsesquiterpene (C16; mw 218) found in Jacobina, Bahia State, or (*S*)-9-methylgermacrene-B [21], a novel monocyclic methylsesquiterpene (C16; mw 218) that is widely distributed throughout Brazil but typically represented by *L*. *longipalpis* from Lapinha Cave, Minas Gerais State. The other two members of the complex produce novel diterpenes, cembrene-1 and cembrene-2 (C20; mw 272) and are represented by the Sobral-2S population from Ceará State and the Jaíbas-1S population from Minas Gerais State [22]. Two of these chemotype populations, (*S*)-9-methylgermacrene-B and cembrene-1, have been identified in São Paulo State [23].

Considering the remarkable epidemiological differences between the two municipalities of Araçatuba and Espírito Santo do Pinhal (mainly the number of human cases notified as well as the abundance, and chemotype of the *L*. *longipalpis* population present in each urban area), Casanova et al (2006) [23] suggested that the (S)-9-methylgermacrene-B and cembrene-1—chemotype populations had different vectorial capacities. If this is true then it is important to have more detailed information on the distribution of the chemotypes to produce more accurate risk maps and to direct more effective control programs. With this in mind, the present study is aimed at determining the chemotypes of a greater number of *L*. *longipalpis* populations from different regions of São Paulo State.

## Methods

### Study area

**Fig 1:**
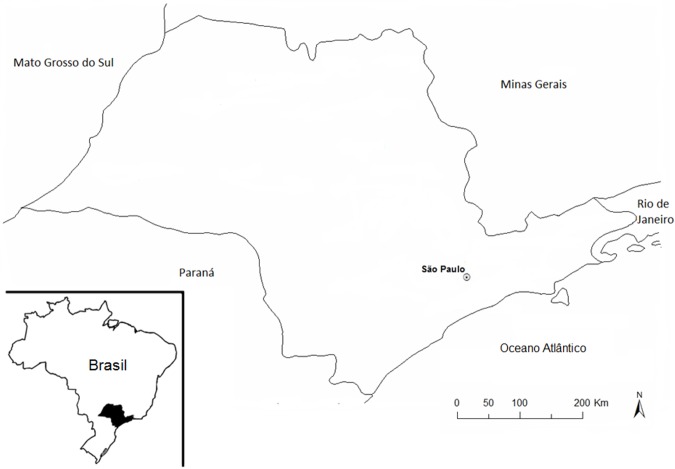
Study area. São Paulo and neighboring states.

São Paulo State is located in Southeast region of Brazil, and shares borders with Minas Gerais to the North and Northeast, Paraná to the South, Rio de Janeiro to the East and Mato Grosso do Sul to the West, and to the Southeast, the Atlantic Ocean (Fig. 1). It is divided into 645 municipalities totalling 248,209 km^2^.

Its climate can be divided into seven distinct types, most are classified as humid. According to Koeppen’s climate classification, the predominant climate type is Cwa, which includes Central and Eastern São Paulo, defined as high-altitude tropical climate, where summer is the rainy season, winter is dry, and the average temperature in summer is above 22°C. In the West region (Aw climate type), the rainy season is delayed until autumn, the winters are dry (the precipitation for the driest month is less than 60mm) and the average temperature for the coldest month is above 18°C [24].

### Human cases

In Brazil, including São Paulo State, American visceral leishmaniasis is a compulsory notifiable disease. Data used in the present study were obtained from Epidemiological Surveillance Centre of Secretary of Health of São Paulo State [13]

### Canine cases

Since the beginning of visceral leishmaniasis surveillance and control activities in São Paulo State, all the municipalities are expected to notify the first- confirmed (laboratory based parasitological identification) occurrence of *L*. *i*. *chagasi*. Data were obtained from canine surveys carried out by the municipalities, Adolfo Lutz Institute and the Secretary of Health of São Paulo State.

### *Lutzomyia longipalpis* data

*L*. *longipalpis* distribution data was obtained from both published data and, principally, from entomological collections carried out by Secretary of Health of São Paulo State, during the performance of their epidemiologic surveillance activities [8, 11, 23, 25–27]. These activities included annual or biannual collections, with CDC light traps, in a minimum of 4 dwellings (more where possible) of all the municipalities considered silent—i.e. without canine or human cases, non-receptive—i.e. where *L*. *longipalpis* has not yet been found—and those considered vulnerable—i.e. those municipalities that are located near to or that connected because of intense transportation of goods and people by road and railway with municipalities with transmission [25]. Annual entomological collections were also carried out in at least 42 dwellings in areas where proven or suspected human or canine transmission occurs but where the vector has not yet been registered.

### Chemotype populations

Male *L*. *longipalpis* from different municipalities were collected manually with an aspirator or CDC electric light trap from peridomiciliary habitats within urban, peri-urban and rural areas, always with permission from local homeowners. The attempts to collect males were made in at least three evenings in four peridomicilies of each sampled municipality. Samples from western São Paulo State were obtained in 11 municipalities that were selected so as to represent all the vector distribution area. For the eastern area, where only 25 municipalities have registered the presence of the vector, nine municipalities, including those with canine transmission were sampled. The sampled municipalities, the geographic coordinates, the number of males chemically analysed and the collection year were respectively: Araçatuba (21°12'14” S; 50°42'61” W), >100, 2005 and 2009; Promissão (21°32'18'' S; 49°51'28'' W), 35, 2009; Bauru (22°18'55'' S; 49°03'41'' W), 23, 2009; Dracena (21°29'00'' S; 51°32'01'' W), 22, 2009; Adamantina (21°40'32'' S; 51°03'47'' W), 13, 2011; Oswaldo Cruz (21°47'38'' S; 50°53'08'' W), 17, 2011; Jales (20°16'06'' S; 50°32'56'' W), 10, 2013; Presidente Prudente (22°07'39'' S; 51°23'08'' W), 13, 2010; Marília (22°13'15'' S; 49°56'55'' W), 9, 2012; Salmourão (22°13'15'' S; 49°56'55'' W), 2, 2013; Lourdes (20°58'01" S; 50°13'27" W), 2, 2013; Espírito Santo do Pinhal (22°10'60'' S; 46°45'45'' W), >20, 2004 and 2009; Socorro (22°35'50'' S; 46°31’04'' W), 1, 2012; Salto (23°12'10'' S; 47°17'11'' W), 2, 2012; São Pedro (22°36'00'' S; 47°52'31'' W), 28, 2009; Indaiatuba (23°5'18'' S; 47°13'24'' W), 3, 2012; Campinas (22°54'23'' S; 47°03'42'' W), 10, 2009; Águas da Prata (21°56'18'' S; 46°42'54'' W), 4, 2013; Sorocaba (23°30'22'' S; 47°27'21'' W), 13, 2013; Votorantim (23°32'26'' S; 47°26'38'' W), 5, 2013. All males were observed under a stereomicroscope to identify to the species level by examination of external morphological characteristics (pale spots on the 4th or 3rd and 4th abdominal tergites and a pair of spikes on the paramere).

All males were killed by placing them in a freezer at-20° C for 10 minutes. They were then placed individually in a glass vial and then covered with hexane (ca. 20 μl). Analysis of male sex pheromone extracts was on a HP-5MS capillary column, 30 m x 0.25 mm i.d., 0.25 μm film thickness (Agilent, Stockport, Cheshire) in a Hewlett Packard 5890 II+ Gas Chromatograph coupled to a Hewlett Packard 5972A bench-top mass spectrometer (electron impact, 70 eV, 180°C). Injection and chromatography conditions were as previously described [19].

## Results

**Fig 2:**
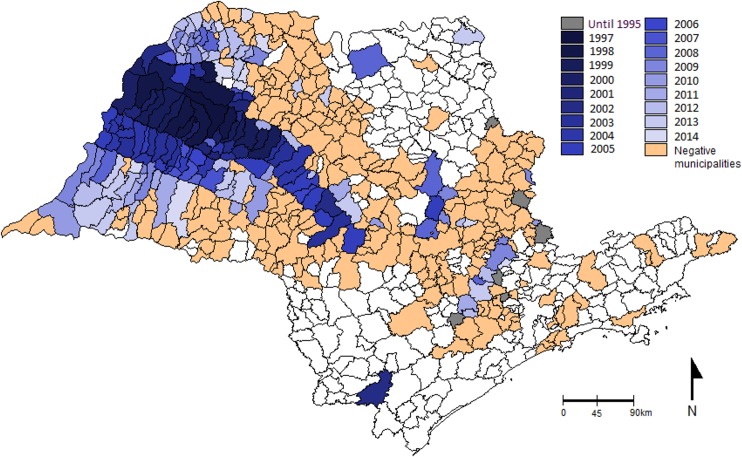
Expansion route of *Lutzomyia longipalpis*. Distribution of *Lutzomyia longipalpis*, in municipalities of São Paulo State, according to the first record, from the 1970’s to June 2014. In negative municipalities, the entomological collections have not recorded the presence of *L*. *longipalpis*.

**Fig 3:**
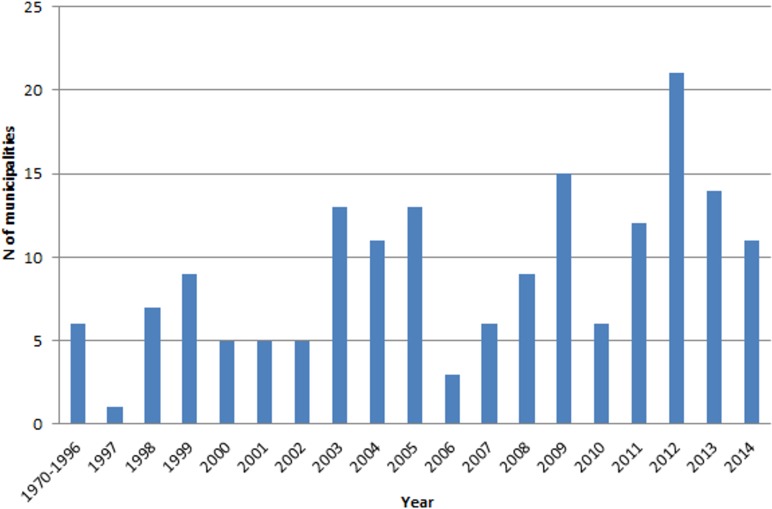
Annual new records of *Lutzomyia longipalpis*. Annual distribution of municipalities which reported *Lutzomyia longipalpis* for the first time in São Paulo State, from 1970’s to June 2014.

Before 1997, *L*. *longipalpis* had been found only in the rural areas of six municipalities of São Paulo State, all of which are in the East and Northeast regions of the state. The first report of the vector in an urban area was in 1997 in the municipality of Araçatuba, in the Western region near the border with Mato Grosso do Sul State (Fig. 2). From 1998 to June 2014, *L*. *longipalpis* has been reported in another 164 municipalities (Fig. 2, S1 Database). During this period, between 2 and 21 new municipalities per year reported the presence of *L*. *longipalpis*, with more than 45 reporting the presence of the vector in the last 3 years (Fig. 3). The biggest expansion in the distribution of *L*. *longipalpis* happened in the western part of Sao Paulo where 146 municipalities have recorded their presence in urban areas during this 17.5 year period.

**Fig 4:**
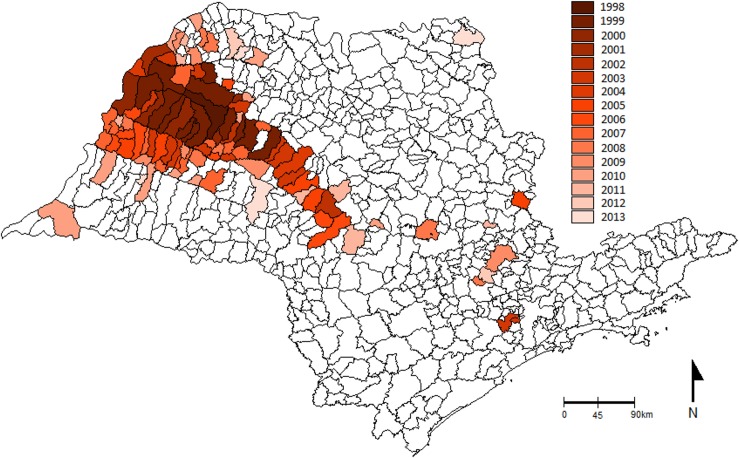
Expansion route of canine cases. Distribution of American visceral leishmaniasis in São Paulo State, according to the record of the first canine case, from 1998 to 2013.

**Fig 5:**
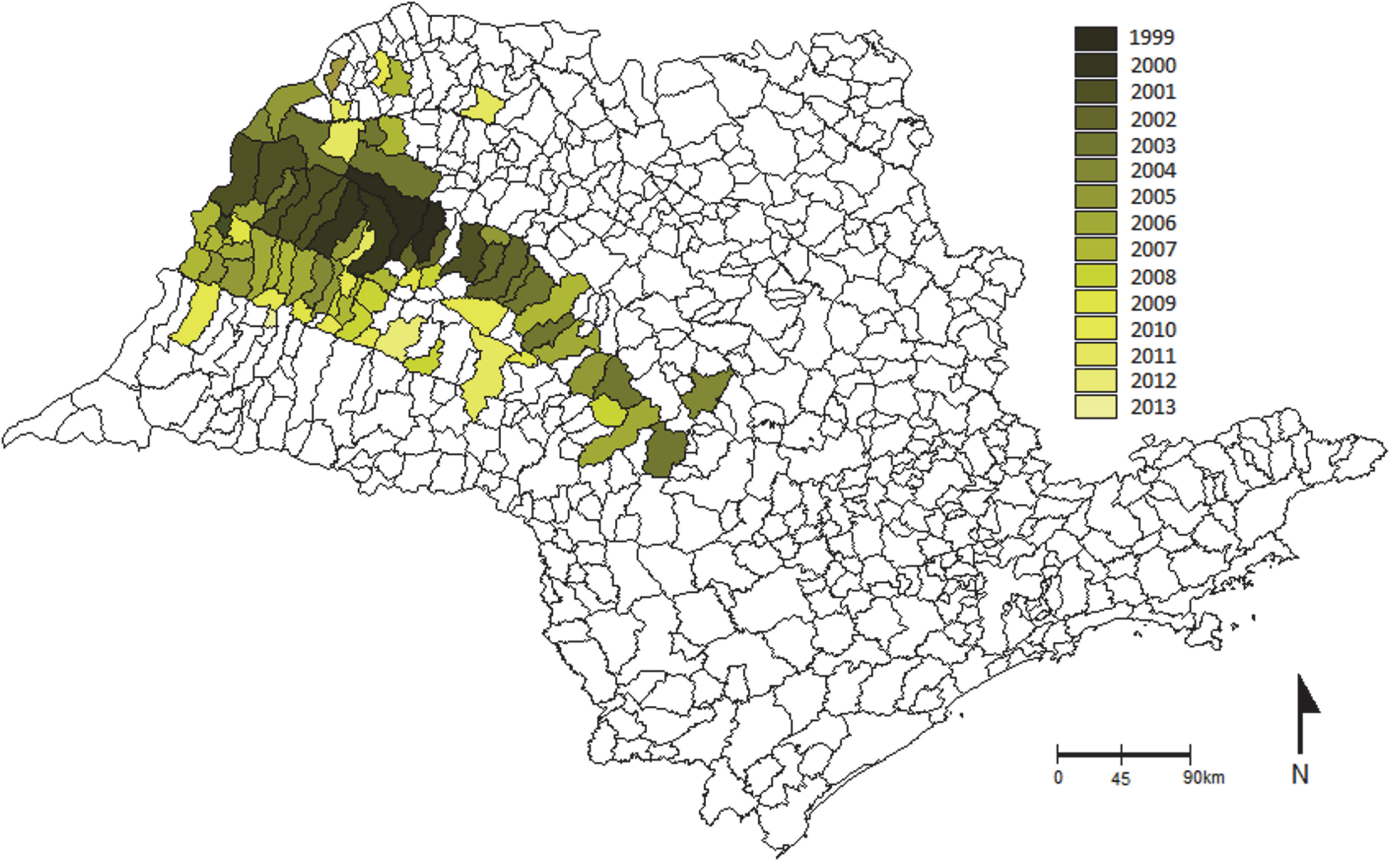
Expansion route of human cases. Distribution of American visceral leishmaniasis in São Paulo State, according to the record of the first human case, from 1999 to April 2013.

The spatial and temporal distribution of *L*. *longipalpis* and human and canine cases in general shows that the presence of the vector preceded the canine cases, and these in turn preceded the human cases (Figs. 2, 4, 5, S1 Database). Up until 2014, there have been 105 municipalities with canine and/or human VL transmission. The majority of these municipalities (93.3%), are placed in the Western part of São Paulo State, and the cases show an expansion route in a Southeasterly direction, towards the Central region, and from there, an expansion both to the North and the South (Figs. 4 and 5). In 71 of these municipalities, there has been both human and canine cases, in 23 only canine transmission and in five only human cases (Figs. 4 and 5). It is interesting to note that in the East region, *L*. *longipalpis* has been found in 25 municipalities only, with canine cases reported in seven, and no known human cases.

**Fig 6:**
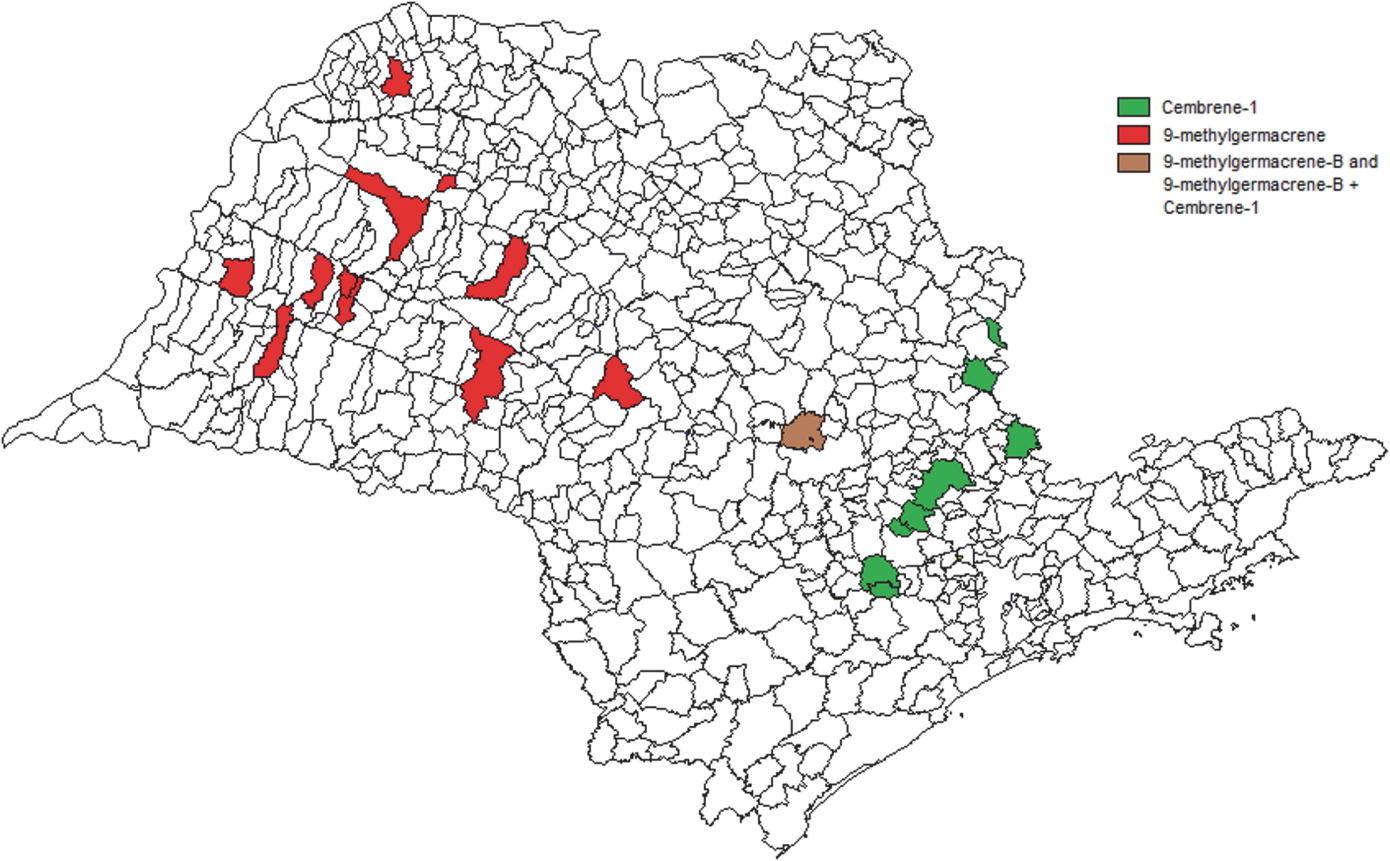
Spatial distribution of chemotype populations. Distribution of (*S*)-9-methylgemacrene-B and cembrene-1 pheromone producing population in São Paulo State. Map shows only municipalities sampled for chemical analysis.

The chemical analysis of all samples of *L*. *longipalpis* males from 11 municipalities in the West region (Araçatuba, Promissão, Bauru, Dracena, Adamantina, Oswaldo Cruz, Jales, Presidente Prudente, Marília, Lourdes and Salmourão) have been shown to contain (*S*)-9-methylgermacrene-B (Fig. 6). On the other hand, all samples of male *L*. *longipalpis* collected in eight municipalities of the Eastern region (Espírito Santo do Pinhal, Socorro, Salto, Indaiatuba, Campinas, Águas da Prata, Sorocaba and Votorantim) contained cembrene-1 (C-20) (Fig. 6). In the municipality of São Pedro, situated in Central region of the State, it was found a (*S*)-9-methylgermacrene-B producing population and in addition, two flies that produced both (*S*)-9-methylgermancrene-B, and cembrene-1.

## Discussion

The argument in favour of the hypothesis of the recent introduction of *L*. *longipalpis* into the Western region of São Paulo State can be supported by its absence, for decades, from various sporadic rural collections of sand flies [28–30]. These collections were done in areas where autochthonous cases of cutaneous leishmaniasis, caused by *Leishmania (Viannia) braziliensis*, had been reported. The contrary hypothesis, that *L*. *longipalpis* has always been there, hidden in the primitive natural vegetation habitat, could be supported because of the existence of several areas where collections have never been done [28–30] Therefore, there are gaps in our knowledge of its distribution and in future, it would be interesting to collect samples from the few natural vegetation areas of São Paulo State.

It is very difficult to pinpoint the year when *L*. *longipalpis* first reached the urban areas of the municipalities in the Western region, however it is likely that, when detected for the first time in the municipality of Araçatuba, in 1997 [8], *L*. *longipalpis* was already present in the urban areas of surrounding municipalities, as the species was found there in the first collections of the following year. From that point on, the spatial and temporal distribution leaves no doubt as to the west-east progression (as far as the central region of the state). This progression can be inferred from the annual urban entomological collection results, which showed that in various municipalities the vector detection only occurred after successive annual negative collections. It is possible that factors related to the economic development of the country, such as the increase in transportation of goods and people by road and railway, could have been responsible for the dispersion of the vector and, consequently, the expansion in the vector’s range in the West.

The higher number of municipalities from the West of the state which reported the presence of *L*. *longipalpis* for the first time since 1997 indicates a rapid inter-municipality dispersion rate. The greatest number of municipalities reported in 2012 and 2013 (21 and 14, respectively) is a further indication of this ongoing, fast expansion. The fact that the expansion of canine and human cases through the Western São Paulo has followed the same dispersion route as that of the vector with a temporal delay cannot be considered to be merely coincidental because it has long been observed that in VL epidemiology the vector precedes canine and subsequently human cases [31]. The lower number of municipalities notifying the presence of the vector in the East region of the state, in contrast to the West, does not show a recognisable dispersion route. Spread of the disease is more likely therefore to be due to the expansion of urban areas into rural or wild areas. This hypothesis is further supported by the observation that *L*. *longipalpis* was only found exclusively in rural habitats in nine of the 25 studied municipalities. In the other 16 municipalities, *L*. *longipalpis* was found in both rural and urban areas in four, and in periurban areas (i.e. those that have the characteristics of rural areas) in 12 municipalities.

It is clear that there are two distinct epidemiological patterns of VL in these two regions of São Paulo State. In the western region it is defined by the occurrence of human cases [12], frequent high prevalence of canine cases [32, 33], and a greater number of municipalities where *L*. *longipalpis* is present. Generally, a great number of flies is frequently found in both manual and CDC light trap collections carried out in peridomiciliary environments associated with food sources, such as chickens and dogs [33, 34]. In this area, all the males analysed have been shown to be the (*S*)-9-methylgermacrene-B chemotype population, including those collected in the 6 of the 18 municipalities currently classified as having moderate or intense transmission in the period from 2010 to 2012. In contrast, the eastern region, can be characterized by the absence of notified human cases—even where the presence of *L*. *longipalpis* and canine cases have been reported for at least 12 years—low prevalence in dogs and a smaller number of municipalities where the vector is present. The populations of sand flies are generally low in abundance in manual and CDC light trap collections carried out in peridomiciliary and rural environments associated with the feeding sources, such as chickens and dogs [35]. All samples of males analysed were the cembrene-1 chemotype.

These observations support the hypothesis of Casanova et al [2006] [23] which proposes that the (*S*)-9-methylgermacrene-B chemotype population has a greater vectorial capacity than the cembrene-1 chemotype in São Paulo State. Differences in ecological parameters of the vector capacity (e.g. vector abundance, survival, host feeding pattern and blood feeding rate) could vary between the two chemotype populations. Furthermore, susceptibility and coevolutionary interactions with *Leishmania* genotypes, which can influence *Leishmania* transmission, are parameters involved in vectorial competence and can vary between different species of *L*. *longipalpis* complex [15, 36, 37]. It is interesting to note that the two main genetic clusters of *L*. *i*. *chagasi*, identified by multilocus microsatellite typing, isolated from dogs from Northwest and Southeast regions of the São Paulo State [38] show distribution coinciding with the two *L*. *longipalpis* chemotype populations distribution presented here. The present study indicates that the chemotype of *L*. *longipalpis* populations as well as the effect of spatial and temporal environmental heterogeneity (reviewed in Belo et al 2013 [39]), should also be considered when explaining the variety of eco-epidemiological transmission scenarios in São Paulo State. However, it is also important to mention that the available data for pheromone types populations from municipalities of other states of Brazil and classified by the Brazilian Ministry of Health as having moderate and intense transmission of VL in the period from 2009 to 2011, shows that in three of these municipalities (Marajó-PA, Natal-RN, Pancas-ES) the chemotype population is cembrene-1, in two (Terezina-PI, Campo Grande-MS) it is (*S*)-9-methylgermacrene-B chemotype, and in one (Sobral-CE) both occur [7, 15]. Further studies are required to access the parameters of vectorial capacity of the longipalpis species complex.

The (*S*)-9-methylgermacrene-B, distributed throughout São Paulo State’s western region has previously been found and characterized in males from Lapinha Cave-MG [21] and later in populations from Sobral-CE, Terezina-PI, Campo Grande-MS, Barra de Guaratiba-RJ, Montes Claros-MG and Araçatuba-SP [15, 19, 23, 40]. The diterpene (C-20) found in populations from municipalities of the East region of the state was previously found and characterized as cembrene-1 in *L*. *longipalpis* from Sobral-CE, and later also found in populations from Marajó-PA, Natal-RN, Estrela-AL, Jaíba-MG, Pancas-ES and Espírito Santo do Pinhal-SP [15, 19, 23]. The presence of a cembrene-1 population in São Paulo State is the southernmost extension of this chemotype.

Based on the results of our present study we suggest that the *L*. *longipalpis* cembrene-1 populations are of rural origin and native of the Eastern region of the São Paulo State, while (*S*)-9-methylgermacrene-B is an introduced chemotype population. Although *L*. *longipalpis* has been found in urban areas of several municipalities of Mato Grosso do Sul State [41], we are not aware of any publications on *L*. *longipalpis* distribution through time in this State. However, although human cases are not a good space-time indicator of parasite circulation, the occurrence of the first human autochthonous case in a municipality usually indicates that the vector and the canine transmission were already established in the area. The West-to-East expansion of human VL in Mato Grosso do Sul was properly demonstrated by Correa-Antonialli et al (2007) [42] who pointed to the construction of the Bolivia-Brazil gas pipeline as a possible cause for the VL time and space expansion. This same hypothesis was considered to explain the spread of the canine and human disease in the west region of São Paulo [12, 38]. Finding the same pheromone type in males in the same temporal expansion route from West to East in São Paulo State may also support the hypothesis that the (*S*)-9-methylgermacrene-B *L*. *longipalpis* chemotype has been introduced from Mato Grosso do Sul.

More samples of males from São Pedro, in the Central region of São Paulo State, should be analysed to allow a better understanding of the possible presence and distribution of sympatric populations of the two pheromone chemotypes. Molecular and behavioural analyses such as those done by Araki et al (2009, 2013) [15, 43] may also help to clarify this question.

Information on the dispersion route and distribution of *L*. *longipalpis* chemotype populations is essential to understand the epidemiological patterns observed in São Paulo State. It may be used to identify areas at risk and consequently define priorities for control measures. In addition, identifying the distribution of the different chemotype populations is helpful in the application of appropriate synthetic male sex pheromone, in pheromone-baited traps or other appropriate “attract-and-kill” approaches [40, 44–49].

## Supporting Information

S1 Database List of municipalities of São Paulo State, according to their epidemiological classification. Record of the first encounter of *Lutzomyia longipalpis* and the first registered canine and human cases.
(XLS)
